# Role of AP-2α and MAPK7 in the regulation of autocrine TGF-β/miR-200b signals to maintain epithelial-mesenchymal transition in cholangiocarcinoma

**DOI:** 10.1186/s13045-017-0528-6

**Published:** 2017-10-30

**Authors:** Dawei Zhang, Haiyan Li, Xiaofeng Jiang, Liangqi Cao, Zilong Wen, Xuewei Yang, Ping Xue

**Affiliations:** 1 Department of Hepatobiliary Surgery, The Second Affiliated Hospital of Guangzhou Medical University, No 250 East Changgang Road, Guangzhou, 510260 China; 2 Department of Breast and Thyroid Surgery, The Sixth Affiliated Hospital of Sun Yat-sen University, Guangzhou, Guangdong 510655 China

**Keywords:** TGF-β, miR-200b, AP-2α, MAPK7, EMT, Cholangiocarcinoma

## Abstract

**Background:**

Cholangiocarcinoma (CCA) is characterized by early lymphatic, metastasis, and low survival rate. Epithelial-mesenchymal transition (EMT) is able to induce tumor metastasis. Although the TGF-β/miR-200 signals promote EMT in various types of cancer, the regulatory mechanism in CCA is still unclear.

**Methods:**

Expression of miR-200b, TGF-β, and EMT markers were measured in tumor samples and cell lines by qRT-PCR and western blot. CCK8 assay was performed to measure the cell viability. Transwell assay was used to evaluate migration and invasion. The target genes of miR-200b and transcription factor of TGF-β were analyzed using dual-luciferase reporter system.

**Results:**

We have demonstrated that CCA exhibited remarkable EMT phenotype and miR-200b was reduced in CCA patients (*n* = 20) and negatively correlated to TGF-β. Moreover, two CCA cells, HCCC, and RBE, with epithelial appearances treated with TGF-β, showed fibroblastic-like cell morphology with downregulated miR-200b expression. Forced expression of miR-200b abrogated TGF-β-induced EMT initiation, with decreased cell proliferation, migration, and invasion in vitro. Also, TFAP2A (encode AP-2α) and MAPK7 were found to be targeted by miR-200b to downregulate EMT and AP-2α inhibited miR-200b by directly promoting transcription of TGFB1. Overexpression of MAPK7 significantly reversed miR-200b-induced inhibition of EMT, migration, and proliferation by increasing the expression of TGF-β, cyclin D1, and Cdk2. Further, the administration of miR-200b induced a remarkably tumor regression in vivo and reduced the effect of TGF-β-related EMT in AP-2α and MAPK7-dependent manner.

**Conclusions:**

Our study highlights that miR-200b-based gene therapy is effective in the treatment of CCA.

## Background

Cholangiocarcinoma (CCA) accounts for 15% of all primary liver cancers and comprises of malignancy arising from the intrahepatic, perihilar, and distal biliary tree [1]. As the second most common primary hepatic tumor, the incidence and mortality of intrahepatic cholangiocarcinoma (iCCA) have increased worldwide during the past decades [1–3].The majority of patients with CCA are often diagnosed at advanced stage, with aggressive behavior, including early lymphatic and metastatic spread. Only 10–15% of the patients with CCA are amenable to potentially curative surgical resection. However, the 5-year survival rate of these patients is only 20–30% due to the high rate of recurrence after surgery [4, 5]. Therefore, the investigations on the mechanism of tumor cell dissemination in patients with CCA are necessary for identifying potential therapeutic targets to prevent CCA recurrence.

Epithelial-to-mesenchymal transition (EMT) is a reversible process in which the epithelial cells lose their cell polarity and cell-cell adhesion and acquire mesenchymal features [6, 7]. During carcinogenesis, EMT enables tumor cells to become invasive via downregulation of epithelial-specific marker, including E-cadherin, thyroid transcription factor 1 (TTF-1) and ZEB and upregulation of mesenchymal markers, including Vimentin, α-SMA, and N-cadherin [8]. Mounting evidences indicated that tumor microenvironment harbored aberrant TGF-β expression activated the expression of transcription factor Slug/Snail2, ZEB1, and ZEB2, and contributed to the initiation of EMT [9]. In CCA cells, the TGF-β1 stimulation induced the decreased expression of epithelial markers along with an increased expression of mesenchymal markers [10]. In CCA patients, EMT markers could be used as biomarkers of poor outcome [5] its target therapy in vivo needs further investigations.

MicroRNAs (miRNAs) are small non-coding, endogenous RNAs comprising of 22 nucleotides that negatively regulate gene expression at the post-transcriptional level by targeting the complementary sites in 3-untranslated regions (3-UTRs) of target mRNAs, leading to a translational inhibition or deregulation of target mRNA to participate into carcinogenesis [11, 12]. The miR-200 family, including miR-200a, miR-200b, miR-200c, miR-141, and miR-429, suppresses metastasis in EMT by targeting and reciprocally repressing ZEB1 to regulate EMT and invasion in cancer cells [13–15]. Gregory et al. reported that prolonged autocrine TGF-β signaling promoted reversible DNA methylation of the miR-200 family promoters, which inhibited the expression of miR-200b to abrogate the ZEB/miR-200 balance [16]. In addition, in esophageal squamous cell carcinoma, miR-200b suppresses the integrin β1-AKT pathway by targeting Kindlin-2 to inhibit tumor cell invasiveness [17]. So far only fewer miRNAs have been found to regulate EMT in CCA including miR-221, mir-200c, miR-34a, and miR-21 [5, 10, 18, 19]. In HCCC, a CCA cell-line with epithelial appearances, miR-200c was overexpressed and suppressed EMT by targeting mesenchymal gene expression (ZEB1/2, Vimentin and N-cadherin) [18]. TGF-β expression is increased in CCA and is significantly correlated with lymph node metastasis, distant metastasis, and tumor recurrence [20]. However, the impact of TGF-β/miR-200b signals in CCA and its regulatory mechanism are unclear.

In this study, it was observed that miR-200b was decreased during carcinogenesis with increased expression of TGF-β and was initiated EMT in patients with CCA. Several studies have demonstrated an association between aberrant activator protein-2 (AP-2) and protein kinase MAPK7, which is critical for the malignant transformation in EMT [21, 22]. Further, miR-200b could target transcription factors, AP-2 alpha (TFAP2A), and MAPK7, which, in turn, inhibit the expression of TGF-β, impair TGF-β-induced EMT and decrease cancer cell proliferation in vitro. The anti-tumor ability of miR-200b in vivo indicated its efficiency as potential treatment strategy in the treatment of CCA.

## Methods

### Tumor samples, cell lines, and reagents

Cholangiocarcinoma samples (*n* = 20) including tumor and paracancerous tissues were collected from The Second Affiliated Hospital of Guangzhou Medical University, China. All the slides of cholangiocarcinoma tissues were evaluated by two pathologists according to WHO classifications. All the retrospective specimens were made anonymous according to the ethical and legal standards. This study was approved by the Research Ethics Committee of The Second Affiliated Hospital of Guangzhou Medical University, China. Written informed consent was obtained from all of the patients. The HCCC and RBE cells were purchased from the cell bank of the Chinese Academy of Sciences (Shanghai, China) and were cultured in Dulbecco’s modified Eagle’s medium (DMEM) supplemented with 10% FBS (Life Technologies, USA), 10% ampicillin, and streptomycin at 37 °C, 5% CO2 conditions. Oligonucleotide sequences of miR-200b mimics (5′-UAAUACUGCCUGGUAAUGAUGA-3′) or negative control (5′-UUCUCCGAACGUGUCACGUTT-3′) was purchased from RiboBio (Guangzhou, china). For expression of TFAP2A and MAPK7, the overexpression vector pcDNA3.0-TFAP2A and MAPK7 were conducted by GenePharma (Shanghai, China). Reporter plasmid of full-length 3′-UTR (wild-type or mutant) of TFAP2A and MAPK7 mRNA and pGL-3-TGFB1 was conducted by GenePharma (Shanghai, China). All constructs were finally confirmed by sequencing. E-cadherin, TTF-1, fibronectin, a-SMA, AP-2α/MAPK7, cyclin D1, and CDK2 antibodies were obtained from Cell Signaling Tech (Denver, MA, USA) and Abcam (USA).

### Cell transfection

The HCCC and RBE cells were cultured to about 80% confluence in 6-well plates and, using Lipofectamine 2000 (Invitrogen, USA), the cells were transfected with miR-200b, pcDNA3.0-TFAP2A, and MAPK7 and pGL-3-TGFB1 according to the manufacturer’s instructions. After transfection for the indicated time, the cells were harvested for further experiments.

### CCK-8 assay

After the transfection for indicated time, HCCC and RBE cells were harvested and washed with PBS and cell counting kit-8 (Kumamoto, Japan) mixed with DMEM was used for cell viability assay. The absorbance was measured at 450 nm by a microplate reader (BioTek, Synergy™ HTX, USA).

### Transwell assay

In the migration assay, 2.5 × 10^4^ HCCC and RBE cells were cultured in the upper chamber of a non-coated Transwell insert. Medium (600 μL) supplemented with 10% bovine serum was used as a chemo-attractant to encourage cell migration in the lower chamber. For invasion assay, the upper chamber of the Transwell inserts were coated with 50 μL of 2.0 mg/mL Matrigel, and 5 × 10^4^ HCCC and RBE cells were plated simultaneously in the upper chamber of the Matrigel-coated Transwell insert. The cells were incubated for 24 h; cells that did not migrate or invade were removed using a cotton swab. Using crystal violet staining, the cells were stained and counted under an inverted microscope. Five random views were selected to count the cells. All the independent experiments were repeated thrice.

### Real-time PCR

Quantitative real-time RT-PCR (qRT-PCR) was performed according to the standard protocol, and the expression levels of miR-200b, E-cadherin, TTF-1, fibronectin, and a-SMA were normalized to GAPDH for gene expression. The primers used are listed below:

**Table:** 

Gene	Primer (5′-3′)
*hsa-miR-200b-F*	ACACTCCAGCTGGGTAGCACCATTTGA
*hsa-miR-200b-R*	TGGTGTCGTGGAGTCG
*U6F*	CTCGCTTCGGCAGCACA
*U6 R*	AACGCTTCACGAATTTGCGT
*GAPDH-F*	ACACCCACTCCTCCACCTTT
*GAPDH-R*	TTACTCCTTGGAGGCCATGT
*E-cadherin-F*	ATGGCTTCCCTCTTTCATCTC
*E-cadherin-R*	ATAGTTCCGCTCTGTCTTTGG
*TTF-1-F*	CTCCCCAGGAGTTAAAAGAG
*TTF-1-R*	CATAGTAGATACGCCGACCA
*Fibronectin-F*	CGGTGGCTGTCAGTCAAAG
*Fibronectin-R*	AAACCTCGGCTTCCTCCATAA
*α-SMA-F*	CCCTTGAGAAGAGTTACGAGTTG
*α-SMA-R*	TGATGCTGTTGTAGGTGGTTTC
*TFAP2A-F*	AGGTCAATCTCCCTACACGAG
*TFAP2A-R*	GGAGTAAGGATCTTGCGACTGG
*MAPK7-F*	GGTGACTTTGGTATGGCTCGT
*MAPK7-F*	CCAGAGGTCAATAGCCTGTGTA
*TGFB1-F*	AGCAACAATTCCTGGCGATAC
*TGFB1-R*	CAACCACTGCCGCACAACT

### Western blotting

The total proteins were extracted and dissolved in SDS-PAGE loading buffer, were separated on 4–15% polyacrylamide gel, and were transferred to nitrocellulose membranes (Amersham Biosciences) according to the manufacturer’s instructions. The membranes were blocked in 5% non-fat milk in tris-buffer saline containing 0.1% Tween-20 (TBST) buffer for 1 h at room temperature and were subsequently incubated with primary antibodies overnight at 4 °C. After washing with TBST buffer, the blots were incubated with HRP-conjugated secondary antibody for 1 h at room temperature and were visualized using the ECL-Plus reagent (Millipore, Billerica, MA, USA). GAPDH was used as the loading control in the Western blotting.

### Dual-luciferase assay

To investigate the targets of miR-200b or the role of AP-2α on the transcription of TGFB1, the HCCC cells were co-transfected with 200 ng firefly Luciferase vector, 40 ng Renilla luciferase pRL-TK vector (Promega, USA) and psiCHECK2-UTR (wild-type or mutant) of TFAP2A and MAPK7 and pGL-3-TGFB1 (wild-type or mutant), pcDNA3.0-TFAP2A or miR-200b. Luciferase activity was determined using the dual-luciferase reporter assay system (Promega, USA). Firefly luciferase acted as a reporter gene and Renilla luciferase as a normalized control. The primers used are listed below:

**Table:** 

Gene	Primer (5′-3′)
TFAP2A-WT-XhoI F	AGCCAAAAGATGATGACAAC
TFAP2A-WT-NotI R	TTCTTCATGTGGTTCATGGT
TFAP2A-MUT-F	GACAACATTTTTATTTCATCGGTGAATAAACTTGAAC
TFAP2A-MUT-R	GTTCAAGTTTATTCACCGATGAAATAAAAATGTTGTC
MAPK7-WT-XhoI F	CCCTCGAGGCTCGGCTTGGATTATTCT
MAPK7-WT-NotI R	ATTTGCGGCCGCTTCACAGGCCTCATTTTG
MAPK7-MUT-F	CCCTGAACAATCCTTTTACCGCCGTTATTTTTATTATTAT
MAPK7-MUT-R	ATAATAATAAAAATACGGCGGTAAAAGGATTGTTCAGGG
TGF-β-WT-KpnI-F	GGGGTACCTGTTGACAGACCCTCTTCTCCTACCTTG
TGF-β-XhoI-R	CCGCTCGAGGCTGGGCCACCGTCCTCATCT
TGF-β-MUT-F	GGTGCCCGCCCCCTTAAAGTTCAACGAGAAGGGACCCCCCTC
TGF-β-MUT-R	GAGGGGGTCCCTTCTCGTTGAACTTTAAGGGGGCGGGCACC

### Electrophoretic mobility shift assay (EMSA)

The DNA-binding activity of AP-2α was determined by EMSA using nuclear extracts prepared by a nuclear extract kit (Active Motif; Rixensart, Belgium) from different groups of cells according to the manufacturer’s instructions. The related probes were synthesized (Invitrogen, USA) and incubated with nuclear extracts in the reaction solution. Unlabeled wild-type (site: GCCCCCTTTCCCCCAGGGCTGAAGGGACC) and mutant (site: GCCCCCTTAGGGGGTCCCGAGAAGGGACC) double-stranded competitor oligonucleotides were added to the respective reactions.

### Tumor model

To investigate the tumor suppressive role of miR-200b in vivo, 3 × 10^6^ HCCC cells were subcutaneously injected in rear flank of nude mice (male BALB/c-nu/nu mice, four per group). The miRNA delivery system (micr*ON*™ miRNA agomir and micr*OFF*™ miRNA antagomir) was purchased from RiboBio (Guangzhou, China). For delivery of cholesterol-conjugated RNA, the miR-200b mimics or negative control (10 nM per injection) were delivered via intra-tumoral injection for six times, 3 days apart.

### Statistical analyses

The Statistical Package for Social Sciences version 16.0 (SPSS 16.0, SPSS Inc., Chicago, IL, USA) and the Prism statistical software package (Version 5.0, Graphpad Software Inc.) were used to performed the statistical analyses. Unpaired *t* tests or Mann-Whitney *U* tests were used to compare the two groups, and multiple group comparisons were analyzed with one-way ANOVA. *P* < 0.05 was considered statistically significant. All experiments were performed in triplicates.

## Results

### Correlation between miR-200b and TGF-β-related EMT in CCA patients

**Fig. 1:**
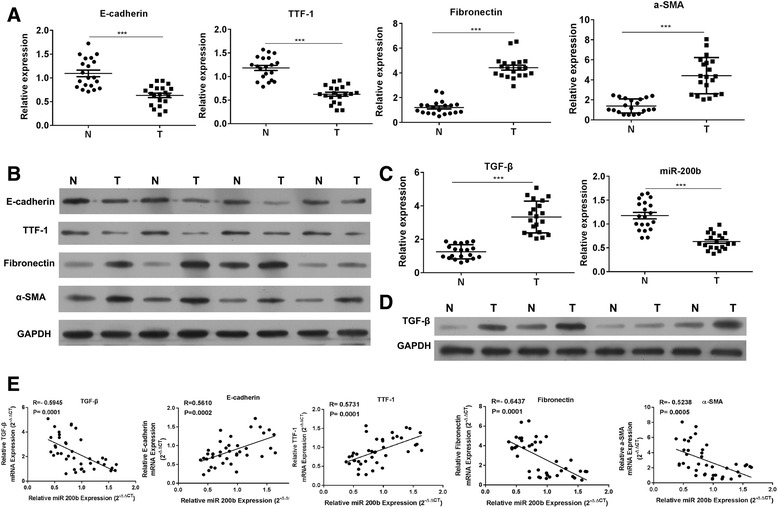
miR-200b is downregulated in CCA patients. **a-b** The mRNA and protein expression of E-cadherin, TTF-1, fibronectin, and α-SMA was determined in CCA tissues by Q-PCR and western blot. **c-d** The mRNA expression of TGF-β and miR-200b was determined in CCA tissues by Q-PCR. **e** The correlation between miR-200b and TGF-β was analyzed; the correlation between miR-200b and EMT markers were analyzed. **p < 0.01, ***p < 0.001, data represent the mean ± SD

Since the majority of patients with CCA are ineligible for curative surgery due to the presence of metastases at the time of diagnosis, it is imperative to confirm the status of EMT in CCA samples prior to further investigations on the mechanism. CCA tissues and corresponding non-neoplastic tissues (*n* = 20) were collected, and the expression of EMT markers, including epithelial-specific marker, E-cadherin, thyroid transcription factor 1 (TTF-1), and mesenchymal markers, including fibronectin and α-SMA, were determined. The results from Q-PCR and western blot indicated that the mRNA expression of E-cadherin and TTF-1 was downregulated, while the expression of fibronectin and α-SMA was increased in CCA tissues (Fig. 1a, b), which indicated that during the progression of CCA, the tumor cell exhibited activated MET phenotype. TGF-β/miR-200b-dependent signaling is a pivotal inducer of EMT in various cancers, such as CCA. It was observed that miR-200b expression was decreased in CCA tissues as compared to non-neoplastic tissues and, conversely, the TGF-β expression was significantly increased in CCA tissues (Fig. 1c, d). Further, correlation analysis indicated that the expression of miR-200b was negatively correlated with the expression of TGF-β, and the decreased miR-200b was associated with the phenotype of EMT by the switch of related markers (Fig. 1e). These findings suggested that the altered expression of miR-200b might be regulated by TGF-β in order to interfere the balance of miR-200b/EMT signals in CCA.

### Effect of TGF-β on the initiation of EMT and inhibition of proliferation

**Fig. 2:**
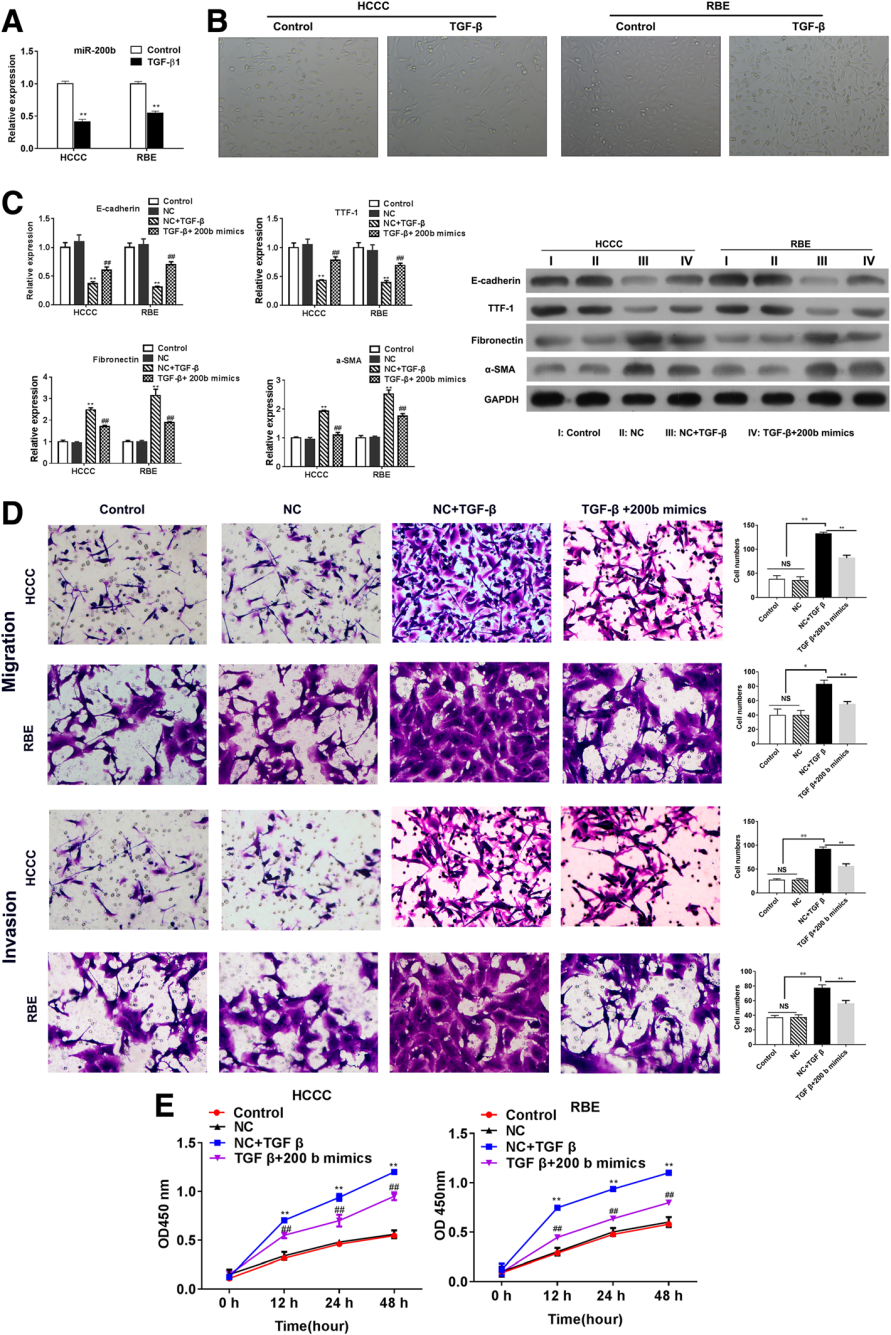
Overexpression of miR-200b inhibits the TGF-β-induced EMT and tumor cell proliferation. **a** The expression of miR-200b was analyzed in TGF-β1-treated HCCC and BRE cells. After the transfection of miR-200b into HCCC and BRE cells, (**b)** the cell morphology was analyzed, (**c**) mRNA and protein expression of E-cadherin, TTF-1, fibronectin, and α-SMA was determined by Q-PCR and western blot. **d** Transwell assay was performed to determine migration and invasion. **e** HCCC and BRE cells were overexpressed with miR-200b, and the cell vitality was measured by CCK-8. *p < 0.05, **p < 0.01, ***p < 0.001, data represent the mean ± SD

To clarify the role of miR-200b in EMT in CAA, two cell-lines, HCCC and BRE, were used in this study. After the stimulation of TGF-β to CCA cell-lines for 2 days, the level of miR-200b was found to be remarkably inhibited (Fig. 2a). Also, a shift from epithelial appearances to fibroblastic-like cell morphology in HCCC and BRE cell lines was observed (Fig. 2b), which significantly increased the ability of migration and invasion of tumor cells. Moreover, we analyzed the mechanism of miR-200b in TGF-β-induced migration and invasion. It was observed that TGF-β repressed the level of E-cadherin and TTF-1 and promoted the expression of fibronectin and α-SMA, which enhanced the ability of migration and invasion of HCCC and BRE cells (Fig. 2c). Overexpression of miR-200b in two cells was demonstrated to abrogate the switch to fibroblastic-like cell phenotype by repressing the expression of fibronectin and α-SMA induced by TGF-β (Fig. 2c). Besides, overexpression of miR-200b inhibit the TGF-β-induced upregulation of migration and invasion (Fig. 2d). The results indicated that high level of TGF-β in tumor microenvironment inhibited the level of miR-200b in CCA tumor cells in order to promote EMT and allow tumor cell metastases.

Further, the HCCC and BRE cells transfected with miR-200b showed decreased cell vitality and proliferation (Fig. 2e), despite the treatment of TGF-β, which suggested that miR-200b could not only inhibit the EMT of tumor cell, but also impair the cell proliferation in CCA.

### Role of TFAP2A in promoting transcription of TGFB1

**Fig. 3:**
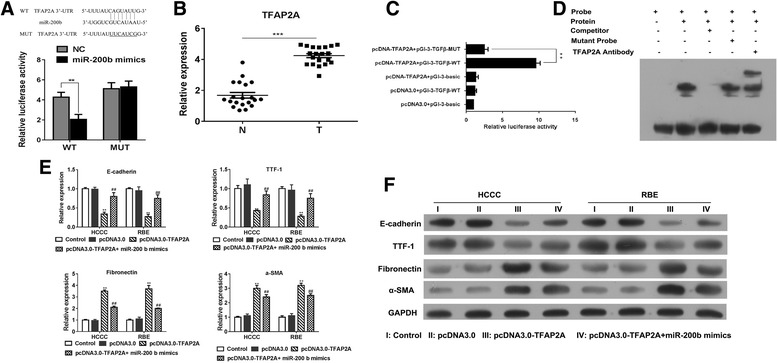
TFAP2A promote transcription of TGFB1 in CCA. **a** The 3′-UTR of TFAP2A exists a binding site of miR-200b; HCCC cell-line was transfected with full-length 3′-UTR (wild-type or mutant) of TFAP2A, and the luciferase reporter was performed to confirm the direct target sites. **b** The mRNA expression of TFAP2A was determined in CCA tissues by Q-PCR. **c** The luciferase reporter was performed to confirm that AP-2α as a transcription factor could promote the transcription of TGFB1. **d** HCCC cells were treated with or without TGF-β and then EMSA experiments were performed to test the ability of AP-2α to directly bind to the TGFB1 promoter. **e-f** HCCC cells were overexpressed with miR-200b and TFAP2A, and the MET phenotype were determined by Q-PCR. *p < 0.05, **p < 0.01, ***p < 0.001, data represent the mean ± SD

We determined the potential targets of miR-200b in TGF-β-induced EMT. According to TargetScan (http://www.targetscan.org/vert_71/), the 3′-UTR of TFAP2A, which encoded AP-2α, harbored a binding site of miR-200b (Fig. 3a). To conform this, a luciferase reporter vector, with full-length 3′-UTR (wild-type or mutant) of TFAP2A, was transfected into HCCC cell-line. The results showed that miR-200b mimics could significantly impair the luciferase activity of wild-type TFAP2A −3’UTR, but not the mutant-type (Fig. 3a). The clinical expression of AP-2α in CCA was also estimated, and upregulated levels of AP-2α was found in tumor samples when compared to normal tissues (Fig. 3b), thereby suggesting that miR-200b could directly target TFAP2A, and downregulation of miR-200b might participate in the tumor metastasis via TFAP2A.

Interestingly, using luciferase reporter assay, we found that AP-2α could promote the transcription of TGFB1 in HCCC cells (Fig. 3c). The data indicated that AP-2α could directly bind to the promoter motif of TGFB1 to induce its expression in autocrine manner, which was also confirmed by EMSA (Fig. 3d).

To provide the evidence that AP-2α participated in miR-200b-regulated EMT in CCA, we transfected miR-200b mimics with AP-2α into the HCCC cells. The results showed that, while miR-200b could prevent the EMT, overexpression of AP-2α significantly increased the level of TGF-β to restore the EMT phenotype of HCCC cell (Fig. 3e, f).

### Effect of MiR-200b on the inhibition of EMT and tumor proliferation

**Fig. 4:**
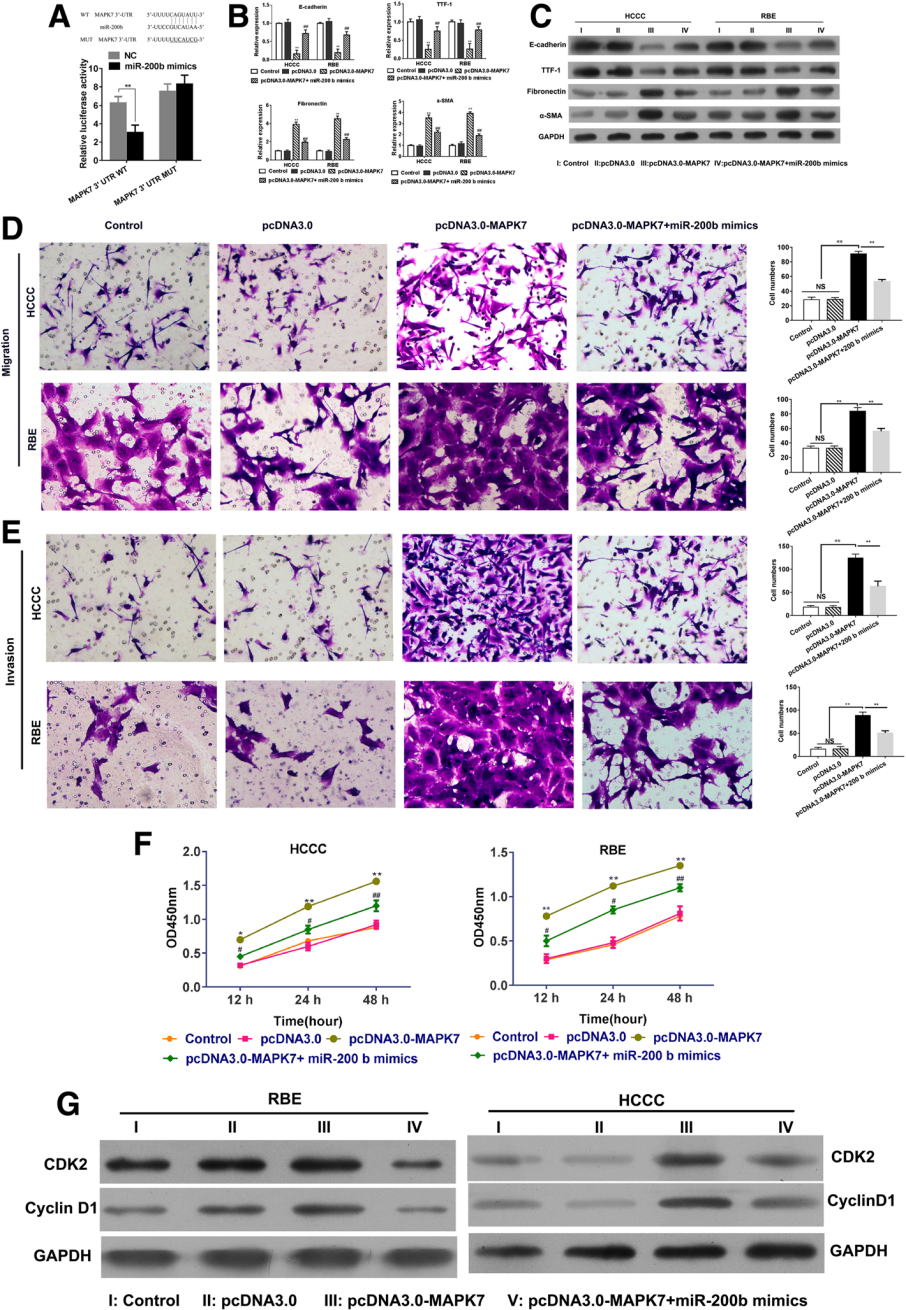
MiR-200b target MAPK7 to inhibit TGF-β-induced EMT and tumor proliferation. **a** The potential binding site of miR-200b in 3′-UTR of MAPK7 was predicted. HCCC cell-line was transfected with full-length 3′-UTR (wild-type or mutant) of MAPK7, and luciferase reporter was performed to confirm the direct target sites. **b-c** HCCC cells were overexpressed with miR-200b and MAPK7 and the mRNA and protein expression of E-cadherin, TTF-1, fibronectin, and α-SMA were determined by Q-PCR and western blot. **d-e** Transwell assay was performed to determine migration and invasion. **f** HCCC cells were overexpressed with miR-200b or MAPK7, and the cell vitality and proliferation were measured by CCK-8. **g** The expression of cyclin D1 and CDK2 were assessed by western blot

MAPK7 belongs to the mitogen-activated protein kinase (MAPK) family of protein kinases and is involved in cell survival, angiogenesis, cell motility, cell proliferation, and EMT [23]. We investigated the function of MAPK7 in TGF-β/miR-200b pathway in CCA. Upon analyzing the 3′-UTR of MAPK7, it was observed that MAPK7 harbored a binding site of miR-200b. Luciferase reporter assays indicated that miR-200b mimics could significantly decrease the luciferase activity of wild-type MAPK7–3′UTR, but not the mutant-type (Fig. 4a), which indicated a direct relationship between miR-200b and MAPK7 in the HCCC cells. Further, overexpression of MAPK7 in HCCC cells could upregulate the TGF-β expression, repress the expression of E-cadherin and TTF-1, and promote the expression of fibronectin and α-SMA in order to re-activate the miR-200b-induced inhibition of EMT (Fig. 4b, c), which was confirmed by the upregulation of migration and invasion (Fig. 4d, e). The results demonstrated a positive regulatory loop between MAPK7 and TGF-β, which regulates EMT via miR-200b in CCA.

We also determined the role of MAPK7 in proliferation and cell cycle. It was observed that MAPK7 played a pivotal role in the cell vitality and proliferation of tumor cells. Forced expression of miR-200b inhibited cell proliferation and vitality (Fig. 4d, e). However, MAPK7 rescued the miR-200b-induced inhibition and increased cell vitality of HCCC cells and promoted the proliferation (Fig. 4f). Mechanistically, overexpression of miR-200b in HCCC cells decreased the expression of cyclin D1 and Cdk2 in order to induce cell cycle arrest and was able restore their expression for the growth of tumor cells (Fig. 4g).

### Correlation between miR-200b and inhibition of EMT and tumor progression in vivo

**Fig. 5:**
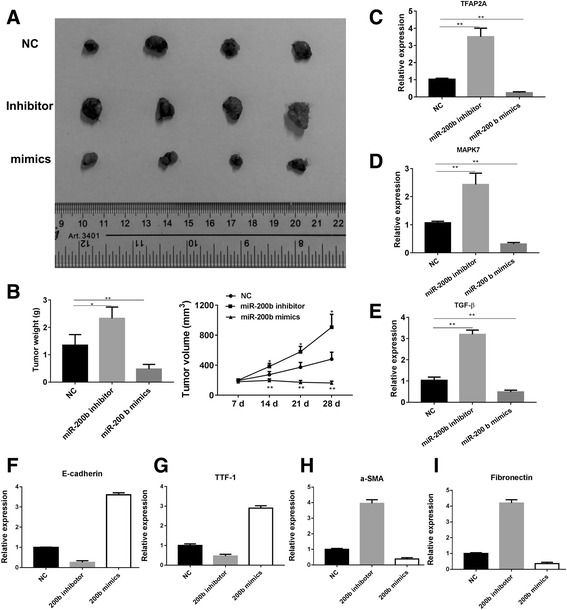
Overexpression of miR-200b prevents EMT and promotes tumor regression. **a-b** Nude mice (six per group) were subcutaneously injected with 3 × 10^6^ HCCC cells and miR-200b mimics, inhibitor or negative control (10 nM per injection) were delivered via intratumoral injection for six times, 3 days apart. The tumor volume (mm^3^) and body weight (g) were measured. **c-d** The level S of AP-2α/MAPK7/TGF-β and EMT markers in tumor tissues was estimated. *p < 0.05, **p < 0.01, ***p < 0.001, data represent the mean ± SD

To determine the role of miR-200b in tumor progression in vivo, two CCA cell-lines, HCCC and BRE, were subcutaneously injected in rear flank of nude mice. The miR-200b mimics, inhibitor or negative control were delivered via intratumoral injection for six times, 3 days apart. The results showed that the mice treated with miR-200b mimics showed a delayed tumor growth, with less tumor weight and volume, than those treated with PBS or negative control (Fig. 5a, b). The status of EMT in tumor samples was analyzed. It was observed that the treatment of miR-200b significantly abrogated the AP-2α/MAPK7/TGF-β expression (Fig. 5c–e), leading to increased expression of E-cadherin and TTF-1 and decreased expression of fibronectin and α-SMA in tumor tissues (Fig. 5f–i). However, the treatment of miR-200b inhibitor promoted the tumor progression with enhanced TGF-β-induced EMT. These findings showed that miR-200b could target AP-2α/MAPK7 to interfere the regulatory loop involving miR-200b and TGF-β in order to repress EMT for cancer metastasis in CCA.

## Discussion

**Fig. 6:**
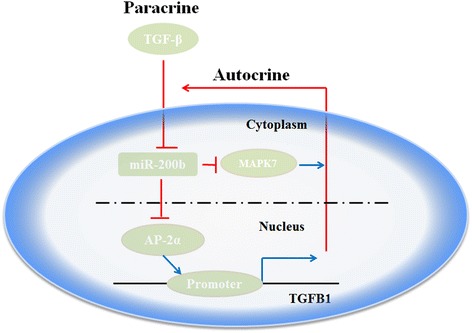
miR-200b/TFAP2A/MAPK7 network regulates the expression of TGF-β1 of and TGF-β1-induced EMT

Despite the curative surgical resection, CCA is associated with high recurrence rate of 50–60%, conferring a 5-year overall survival (OS) of only 20–30% [1]. Increasing evidences have shown that EMT plays the key role in tumor metastatic dissemination [3]. The mechanism of EMT during the CCA, however, is still unclear. In this study, it was observed that CCA demonstrated remarkable EMT phenotype, and miR-200b was reduced in CCA patients (*n* = 20). A regulatory loop involving TGF-β and miR-200b was found to contribute to the maintenance of EMT in CCA via AP-2α and MAPK7. The administration of miR-200b promoted tumor regression in vivo and abolished the maintenance of TGF-β-related EMT in AP-2α-and MAPK7-dependent manner in CCA (Fig. 6).

TGF-β-induced EMT is involved in different pathways, including the NF-휅B signals, MAPK signals, wingless integrated (Wnt) signals, and Notch pathway. miRNA has been found to be the most important mediator in CCA [6, 24]. Several transcription factors, such as Snail, Slug/Snail2, ZEB1, and ZEB2, could repress the transcription of epithelial-specific junction proteins (e.g., E-cadherin) by directly binding to its promoter in order to prevent the initiation of EMT. Siemens H et al. reported that overexpression of miR-34a repressed the level of SNAIL via the miR-34a/b/c seed-matching sequence in the SNAIL 3′-UTR to induced EMT [24]. In human ovarian cancer, miR-125a was found to be a negative regulator of EMT. miR-125a could target the AT-rich interactive domain 3B (ARID3B) to repress the conversion of highly invasive ovarian cancer cells from a mesenchymal to an epithelial morphology [25]. Currently, limited miRNAs are described to regulate EMT in CCA, including miR-221, miR-200c, miR-204, miR-214, miR-34a, and miR-21. In this study, we have demonstrated that the expression of miR-200b was decreased in CCA samples in order to promote the acquisition of EMT phenotype by CCA cells. In addition, ectopic expression of miR-200b could abrogate the switch to fibroblastic-like cell phenotype by repressing the expression of fibronectin and α-SMA induced by TGF-β, and could inhibit the tumor cell vitality and proliferation.

Activator protein-2 (AP-2) participate in the regulation of various different biological factors as transcriptions factors during development, cell growth, differentiation and apoptosis, and carcinogenesis [21]. Although it could function as a tumor suppressor by regulating the expression of various cancer-related processes including cell cycle, EMT and apoptosis [26], the AP-2 family functioned highly cell-type specific in different tissues and many AP-2 activity modulating factors has not been usually analyzed in the AP-2 studies, leading to the discrepancies about the role of AP-2. Campillos M et al. showed that oncogene, DEK, coordinated with AP-2α for the regulation of cell cycle [27]. Zhang et al. reported that overexpression of AP-2α decreased the invasive abilities of BeWo cells by the induction of TIMP-2 and E-cadherin and a significant reduction of MMP-2 and MMP-9 in preeclampsia [28]. In this study, it was observed that AP-2α could directly bind to the promoter motif of TGFB1 in order to induce TGF-β expression, which downregulated miR-200b expression. Since TFAP2A (encode AP-2α) could be directly targeted by miR-200b, decreased miR-200b enhanced AP-2α/ TGF-β signals in CCA for tumor metastasis.

The MEK5/MAPK7 pathway is one of the lesser studied pathways of the mitogen-activated protein kinase (MAPK) family of protein kinases. Inhibition of MAPK7 blocked the TGF-β1 signal for Smad3 transcriptional activity via acetylation and regulated TGF-β1-induced pulmonary fibrosis [29]. In human glioma, MAPK7 was found to be a direct target gene of miR-200b and was essential to the miR-200b-induced inhibition of glioma tumor growth, invasion and EMT [22]. To date, the function of MAPK7 in CCA remains unknown in EMT. In this study, we have demonstrated that the overexpression of MAPK7 in HCCC cells could upregulate the TGF-β expression to re-activate the miR-200b-induced inhibition of EMT and restore the expression of cyclin D1 and Cdk2 to recover cell cycle arrest for the growth of tumor cells.

Importantly, the anti-tumor effects of miR-200 family were demonstrated in different tumor models, including colorectal cancer xenografts models [30], orthotopic glioma, and orthotopic gastric cancer models [31]. In this study, it was showed that the treatment of miR-200b could target AP-2α/MAPK7 in CCA xenografts models in order to interfere the regulatory loop involving miR-200b and TGF-β to repress EMT for cancer metastasis. However, the limitations of CCA xenograft model should be recognized in this study. Immortalized tumor cell lines used in xenograft models have adapted to the situation in vitro that lack of tumor microenvironment of the patient. To some extent, these cell lines lose the primary tumor characteristics, and therefore cannot objectively reflect the situation of primary tumors. Given the advantages in the following aspects, tumor formation rate, lymph nodes metastasis, ascites generation, viscera metastasis, orthotopic transplantation model is more biologically relevant in vivo models and would be used in further study.

## Conclusions

We analyzed the potential mechanism of TGF-β-related EMT in CCA. It was observed that miR-200b was reduced by TGF-β in vitro and in vivo, and downregulated miR-200b significantly increased the expression of its target gene, AP-2α/MAPK7, which promoted the TGF-β level in tumor tissue. These findings showed that the miR-200b-based gene therapy could be conducive in the treatment of CCA patients.

## Abbreviations

CCA: Cholangiocarcinoma
CCK-8: Cell counting kit-8
EMT: Epithelial-mesenchymal transition
MAP K7: Mitogen-activated protein kinase 7
TGF-β: Transform growth factor-β

## References

[1] Charbel H, Al-Kawas FH (2011). Cholangiocarcinoma: epidemiology, risk factors, pathogenesis, and diagnosis. Current gastroenterology reports.

[2] Zhang H, Yang T, Wu M, Shen F (2016). Intrahepatic cholangiocarcinoma: epidemiology, risk factors, diagnosis and surgical management. Cancer Lett.

[3] Chong DQ, Zhu AX. The landscape of targeted therapies for cholangiocarcinoma: current status and emerging targets. Oncotarget. 2016; 10.18632/oncotarget.8775 PMC5216834 27102149

[4] Zhu AX (2015). Future directions in the treatment of cholangiocarcinoma. Best practice & research. Clinical gastroenterology.

[5] Vaquero J, Guedj N, Claperon A, Ho-Bouldoires TH, Paradis V and Fouassier L. Epithelial-mesenchymal transition in cholangiocarcinoma: from clinical evidence to regulatory networks. Journal of hepatology. 2016. 10.1016/j.jhep.2016.09.010 27686679

[6] Nieto MA, Cano A (2012). The epithelial-mesenchymal transition under control: global programs to regulate epithelial plasticity. Semin Cancer Biol.

[7] Mitra A, Mishra L, Li S (2015). EMT, CTCs and CSCs in tumor relapse and drug-resistance. Oncotarget.

[8] Saito RA, Watabe T, Horiguchi K, Kohyama T, Saitoh M, Nagase T (2009). Thyroid transcription factor-1 inhibits transforming growth factor-beta-mediated epithelial-to-mesenchymal transition in lung adenocarcinoma cells. Cancer Res.

[9] Diepenbruck M, Christofori G (2016). Epithelial-mesenchymal transition (EMT) and metastasis: yes, no, maybe?. Curr Opin Cell Biol.

[10] Qiao P, Li G, Bi W, Yang L, Yao L, Wu D (2015). microRNA-34a inhibits epithelial mesenchymal transition in human cholangiocarcinoma by targeting Smad4 through transforming growth factor-beta/Smad pathway. BMC Cancer.

[11] Krol J, Loedige I, Filipowicz W (2010). The widespread regulation of microRNA biogenesis, function and decay. Nat Rev Genet.

[12] Liang D, Xiao-Feng H, Guan-Jun D, Er-Ling H, Sheng C, Ting-Ting W (1852). Activated STING enhances Tregs infiltration in the HPV-related carcinogenesis of tongue squamous cells via the c-jun/CCL22 signal. Biochim Biophys Acta.

[13] Park SM, Gaur AB, Lengyel E, Peter ME (2008). The miR-200 family determines the epithelial phenotype of cancer cells by targeting the E-cadherin repressors ZEB1 and ZEB2. Genes Dev.

[14] Koutsaki M, Spandidos DA, Zaravinos A (2014). Epithelial-mesenchymal transition-associated miRNAs in ovarian carcinoma, with highlight on the miR-200 family: prognostic value and prospective role in ovarian cancer therapeutics. Cancer Lett.

[15] Chatterjee R, Mitra A (2015). An overview of effective therapies and recent advances in biomarkers for chronic liver diseases and associated liver cancer. Int Immunopharmacol.

[16] Gregory PA, Bracken CP, Smith E, Bert AG, Wright JA, Roslan S (2011). An autocrine TGF-beta/ZEB/miR-200 signaling network regulates establishment and maintenance of epithelial-mesenchymal transition. Mol Biol Cell.

[17] Zhang HF, Alshareef A, Wu C, Li S, Jiao JW, Cao HH (2015). Loss of miR-200b promotes invasion via activating the Kindlin-2/integrin beta1/AKT pathway in esophageal squamous cell carcinoma: An E-cadherin-independent mechanism. Oncotarget.

[18] Oishi N, Kumar MR, Roessler S, Ji J, Forgues M, Budhu A (2012). Transcriptomic profiling reveals hepatic stem-like gene signatures and interplay of miR-200c and epithelial-mesenchymal transition in intrahepatic cholangiocarcinoma. Hepatology.

[19] Xie Y, Wehrkamp CJ, Li J, Wang Y, Mott JL, Oupicky D (2016). Delivery of miR-200c Mimic with Poly(amido amine) CXCR4 Antagonists for Combined Inhibition of Cholangiocarcinoma Cell Invasiveness. Mol Pharm.

[20] Sato Y, Harada K, Itatsu K, Ikeda H, Kakuda Y, Shimomura S (2010). Epithelial-mesenchymal transition induced by transforming growth factor-{beta}1/Snail activation aggravates invasive growth of cholangiocarcinoma. Am J Pathol.

[21] Pellikainen JM, Kosma VM (2007). Activator protein-2 in carcinogenesis with a special reference to breast cancer--a mini review. Int J Cancer.

[22] Wu J, Cui H, Zhu Z, Wang L (2016). MicroRNA-200b-3p suppresses epithelial-mesenchymal transition and inhibits tumor growth of glioma through down-regulation of ERK5. Biochem Biophys Res Commun.

[23] Drew BA, Burow ME, Beckman BS (1825). MEK5/ERK5 pathway: the first fifteen years. Biochim Biophys Acta.

[24] Siemens H, Jackstadt R, Hunten S, Kaller M, Menssen A, Gotz U (2011). miR-34 and SNAIL form a double-negative feedback loop to regulate epithelial-mesenchymal transitions. Cell Cycle.

[25] Cowden Dahl KD, Dahl R, Kruichak JN, Hudson LG (2009). The epidermal growth factor receptor responsive miR-125a represses mesenchymal morphology in ovarian cancer cells. Neoplasia.

[26] Su W, Xia J, Chen X, Xu M, Nie L, Chen N (2014). Ectopic expression of AP-2alpha transcription factor suppresses glioma progression. Int J Clin Exp Pathol.

[27] Campillos M, Garcia MA, Valdivieso F, Vazquez J (2003). Transcriptional activation by AP-2alpha is modulated by the oncogene DEK. Nucleic Acids Res.

[28] Zhang Z, Zhang L, Jia L, Cui S, Shi Y, Chang A (2013). AP-2alpha suppresses invasion in BeWo cells by repression of matrix metalloproteinase-2 and -9 and up-regulation of E-cadherin. Mol Cell Biochem.

[29] Kim S, Lim JH, Woo CH (2013). ERK5 inhibition ameliorates pulmonary fibrosis via regulating Smad3 acetylation. Am J Pathol.

[30] Pan Y, Liang H, Chen W, Zhang H, Wang N, Wang F (2015). microRNA-200b and microRNA-200c promote colorectal cancer cell proliferation via targeting the reversion-inducing cysteine-rich protein with Kazal motifs. RNA Biol.

[31] Ning X, Shi Z, Liu X, Zhang A, Han L, Jiang K (2015). DNMT1 and EZH2 mediated methylation silences the microRNA-200b/a/429 gene and promotes tumor progression. Cancer Lett.

